# A priori prediction of breast cancer response to neoadjuvant chemotherapy using quantitative ultrasound, texture derivative and molecular subtype

**DOI:** 10.1038/s41598-023-49478-3

**Published:** 2023-12-19

**Authors:** Lakshmanan Sannachi, Laurentius O. Osapoetra, Daniel DiCenzo, Schontal Halstead, Frances Wright, Nicole Look-Hong, Elzbieta Slodkowska, Sonal Gandhi, Belinda Curpen, Michael C. Kolios, Michael Oelze, Gregory J. Czarnota

**Affiliations:** 1 Physical Sciences Platform, Sunnybrook Research Institute, Sunnybrook Health Sciences Centre, Toronto, ON Canada; 2 Department of Radiation Oncology, Odette Cancer Centre, Sunnybrook Health Sciences Centre, 2075, Bayview Avenue, Toronto, ON M4N 3M5 Canada; 3 Department of Surgery, Sunnybrook Health Sciences Centre, Toronto, ON Canada; 4 Department of Anatomic Pathology, Sunnybrook Health Sciences Centre, Toronto, ON Canada; 5 Department of Medical Imaging, Sunnybrook Health Sciences Centre, Toronto, ON Canada; 6 Department of Physics, Toronto Metropolitan University, Toronto, ON Canada; 7 Department of Electrical and Computer Engineering, Univerity of Illinois Urbana-Champaign, Urbana, IL USA; 8 Department of Medical Biophysics, University of Toronto, Toronto, ON Canada; 9 Department of Radiation Oncology, University of Toronto, Toronto, ON Canada

**Keywords:** Biophysics, Biomarkers

## Abstract

The purpose of this study was to investigate the performances of the tumor response prediction prior to neoadjuvant chemotherapy based on quantitative ultrasound, tumour core-margin, texture derivative analyses, and molecular parameters in a large cohort of patients (n = 208) with locally advanced and earlier-stage breast cancer and combined them to best determine tumour responses with machine learning approach. Two multi-features response prediction algorithms using a k-nearest neighbour and support vector machine were developed with leave-one-out and hold-out cross-validation methods to evaluate the performance of the response prediction models. In a leave-one-out approach, the quantitative ultrasound-texture analysis based model attained good classification performance with 80% of accuracy and AUC of 0.83. Including molecular subtype in the model improved the performance to 83% of accuracy and 0.87 of AUC. Due to limited number of samples in the training process, a model developed with a hold-out approach exhibited a slightly higher bias error in classification performance. The most relevant features selected in predicting the response groups are core-to-margin, texture-derivative, and molecular subtype. These results imply that that baseline tumour-margin, texture derivative analysis methods combined with molecular subtype can potentially be used for the prediction of ultimate treatment response in patients prior to neoadjuvant chemotherapy.

## Introduction

Breast cancer remains a major public health problem for women^1^. Women, particularly those with locally-advanced breast cancer (LABC), have poor long-term survival rates compared to early-stage breast cancer patients^2^. LABC occurs relatively infrequently, but it poses a significant clinical challenge. LABC generally refers to large breast tumours greater than 5 cm, including stage 3–4 disease, and in some cases, will involve the skin and chest wall. LABC may also involve axillary or peripheral lymph nodes. Treatment of LABC and earlier stage but high risk tumours often starts with neoadjuvant chemotherapy (NAC) followed by surgery and then radiation therapy^3,4^. The advantage of neoadjuvant chemotherapy is that it can facilitate breast-conserving surgery in cases where there is a significant reduction in the tumour volume^5^. Along with early diagnosis, optimal therapy management is crucial to reducing mortality from breast cancer. Tumour response is a good prognostic factor for long-term disease-free and overall survival^6^. However, not all patients respond to neoadjuvant chemotherapy equally. At present, breast cancer response to neoadjuvant chemotherapy is assessed from changes in tumour size and histological analysis of the post-operative specimen, which is typically occurs months after staring NAC.

Several studies have reported that patient who do not respond to initial chemotherapy may benefit from salvage therapies, including additional systemic chemotherapy or preoperative radiation or surgery^7–9^. Therefore, finding tumour response biomarkers to NAC prior to treatment, which predict treatment outcomes, is important, as it could facilitate personalized treatment, resulting in improved tumour response to NAC and a better long-term outcome. From a biological point of view, Ki-67, human epithelial growth factor receptor 2, and circulating tumour nucleosomes have been suggested to be predictive of the likelihood of breast tumour response to NAC prior to treatment^10–12^. A previous diffuse optical spectroscopic tomographic image analysis study reported that LABC pathologically complete response patients have significantly higher hemoglobin concentration levels than those with pathologically incomplete response. Recent studies involving CT and MRI images of LABC patients have demonstrated that texture analysis of these images could predict tumour response based on the hypothesize that the microstructure and metabolic characteristics of tumour might be linked to its aggressiveness and responsiveness to NAC^13,14^. Another imaging technique used in LABC tumour response monitoring and prediction is quantitative ultrasound (QUS)^15–18^. Unlike MRI and CT imaging, QUS backscatter-based biomarkers depend on intrinsic contrast alternations arising from changes in the microstructure and elastic properties of cancer cells when they respond to treatment, and hence the methods does not need contrast agents.

The QUS-detected responses in tumours to cancer treatment are related to biological changes in tumour microstructure and spatial inhomogeneity. Based on this hypothesis, QUS has been utilized in several pre-clinical and clinical studies to detect tumour response to treatment early and during a course of treatment^16,19,20^. In those studies, QUS spectral parameters such as mid-band fit (MBF), spectral slope (SS) and 0-MHz intercept (SI) were investigated for evaluating patient responses to chemotherapy and showed a significant correlation with tumour response. These spectral parameters are related to scatterer properties such as scatterer size and scatterer acoustic concentration^17^. Additionally, these scatterer properties; average scatterer diameter (ASD) and average acoustic concentration (AAC) which are determined from the backscatter coefficient by fitting a spherical Gaussian model to the measured backscatter coefficient, have been used for treatment response monitoring in locally advanced breast tumours treated with chemotherapy^16^. In an another study, textural features determined from QUS parametric maps, such as contrast, correlation, energy and homogeneity, have been investigated in tumour response monitoring^16,17,21,22^. These texture parameters, which quantify the spatial relationship between neighboring acoustic property within tissue microstructures, have been demonstrated to be capable of characterizing response heterogeneities^23^. Combining mean QUS values and texture features of the QUS parametric maps in response detection model development has demonstrated improvement in accuracies for monitoring tumour response.

In our previous study, tumour-margin analysis of QUS parametric images acquired from LABC patients was investigated for the prediction of tumour response to neoadjuvant chemotherapy before start of treatment, based on the hypothesis that the margin may account for the presence of microscopic infiltration from the primary tumour into the surrounding normal tissue^24^. The result demonstrated that, in addition to tumour core, QUS analysis of a 5-mm tumour surrounding region improved tumour response prediction performance. Most recently, texture-derivative parameters were derived from QUS parametric maps by constructing GLCM-based texture maps using a sliding window analysis^18^. That study reported further improvement in tumour response prediction before the start of treatment based on hypothesis that the second-order texture-derivatives parameters reflect intra-tumoural heterogeneity better than fundamental textural parameters, leading to improve the prediction of clinical outcomes.

Several investigators have explored the association between breast cancer molecular features and pathological complete response after NAC. In an early-stage breast cancer study, researchers examined the relationship between molecular features and recurrence-free survival, revealing a robust correlation between them^25^. Similarly, in a LABC study, investigators reported that HER2 + and triple negative breast cancer exhibit a higher rate of pathological complete response^12,26^. Other studies have highlighted significant differences in chemotherapy response rates and survival among breast cancer patients with various molecular subtypes, including HER2 + , triple negative, and ER and/or PR + with HER2- status^27^.

The current study builds from previous work^18^ by a significant expansion of the patient cohort involved. Additionally, the feature space is broadened by incorporating QUS, texture and texture-derivate features from the 5-mm tumour margin. In the present study, we examined 208 breast cancer patients including both locally advanced and earlier-stage cancer patients, who received NAC. Patients were categorized into two response groups based on modified response grading system described in our previous study^16^. The correlation of quantitative ultrasound, texture, texture derivative parameters estimated from primary tumour and 5-mm tumour margin ultrasound data with response was investigated. Core and margin analyses were combined with molecular subtype to enhance the prior treatment tumour response prediction model. To develop a highly accurate response prediction model, two standard classification algorithms in machine learning, nearest neighbors (KNN) and a support vector machines-radial basis function (SVM-RBF) were evaluated. We compared the performance of the diagnostic models developed based on margin analysis, texture derivatives, and molecular subtype, and their combination to understand the importance of these feature sets in tumour response. The obtained models were cross-validated using both leave-one-out (LOO) and hold-out cross-validation techniques to investigate the limitations of the dataset in developing tumour response prediction during the training process. Classifier performance was evaluated here using receiver operating characteristics (ROC) analysis to obtain metrics such us sensitivity, specificity, accuracy, area under the receiver operating characteristic curve (AUC), positive predictive value (PPV), and negative predictive value (NPV). This work demonstrates a method to predict tumour treatment response before treatment initiation based on mean values of QUS parameters, texture, texture-derivatives, and tumour molecular subtype, potentially aiding clinicians in personalizing NAC for breast cancer.

## Material and methods

### Study protocol

In this study, 208 breast cancer patients including locally advanced and earlier-stage breast cancer were examined. This study adhered to the appropriate guidelines of the Sunnybrook Research Institute Research Ethics Board at Sunnybrook Health Sciences Centre (SHSC), Toronto, Canada. All experimental protocols were reviewed and approved by the Sunnybrook Research Institute Research Ethics Board at Sunnybrook Health Sciences Centre (SHSC), Toronto, Canada before commencing the study. All patients were enrolled with informed consent. As part of their standard care, all patients underwent a core needle biopsy before treatment to confirm a cancer diagnosis, histological subtype, and hormone receptor status, including estrogen receptor (ER), progesterone receptor (PR), and human epidermal growth factor receptor 2 (HER2)) to determine tumour molecular subtype. Magnetic resonance images were obtained before treatment as part of clinical care to establish the initial tumour size. Ultrasonic data were acquired immediately before patients started chemotherapy. Following mastectomy, the patients’ mastectomy specimens were prepared on a 5˝ × 7˝ whole-mount pathology slide and digitized using a confocal scanner (TISSUEscope™, Huron Technologies, Waterloo, ON). A board-certified pathologist examined the specimens and recorded the results in the patient’s medical chart.

Patients were classified into two groups including responder (R) and non-responder (NR) using a modified response grading system based on the clinical/pathological tumour response determined at the end of their treatment^16^. The response category included the disappearance of all target lesions, and any pathological lymph nodes must have reduction in short axis to < 10 mm or at least 30% decrease in diameter of target lesions or cellularity < 5% in the tumour bed (invasive disease) irrespective of size. This category incorporates both complete responders and partial responders. The non-response category included decrease in tumour size less than 30%, accompanied by no significant changes in tumour cellularity. This category incorporated stable disease and progressive disease.

### Ultrasound data acquisition

All ultrasonic breast imaging and RF data acquisition were performed with a Sonix RP clinical research system (Analogic Medical Corp., Vancouver, Canada). A linear array transducer. L14-5/60 (Analogic Medical Corp., Vancouver, Canada), operating at a central frequency of 6.5 MHz was used, with a bandwidth range of 3–8 MHz and sampling at 40 MHz. The sector size for each image frame was 6 cm (lateral distance) and 4–6 cm (axial depth), storing 512 RF lines across the lateral distance. Four to seven image planes were acquired at 1 cm intervals across the involved breast, with the transducer focus set at the midline of the tumour. Although the majority of tumours were readily visible with ultrasound, tumour location was cross-verified using the patient’s dynamic contrast-enhanced MR images. The regions of interest of tumour were selected manually by a radiologist for all tumour RF-data frames. For tumour-margin analysis, in addition to tumour core, a 5-mm distance surrounding area was selected. The quantitative spectral, texture and texture-derivate analyses were performed on selected ROIs covering the tumour core and 5-mm margin.

### Ultrasound data analysis

The quantitative ultrasound parameters, including MBF (mid-band fit), SS (spectral slope), SI (spectral intercept), ACE (attenuation co-efficient estimate), ASD (acoustic scatterer diameter) and AAC (average acoustic-scatterer concentration) were determined from ROI-selected tumour core and 5-mm margin areas using quantitative ultrasound methods^16^. In this technique, each ROI was divided into window blocks of size 10 times the ultrasound wavelength with 94% overlap in both the axial and lateral directions to construct QUS parametric images. Tumour attenuation was determined using a spectral difference method^28^. The reference phantom method was used to remove any ultrasound system dependencies in quantitative parameters estimation. The attenuation coefficient and speed of sound of the reference phantom were 0.786 dB/MHz/cm and 1540 m/s, respectively. MBF, SS and SI were calculated using linear regression analysis of the normalized backscatter power spectrum over the − 6 dB bandwidth of the transducer^29^. The ASD and AAC parameters were derived from the backscatter coefficient by comparing measured data with a theoretically derived backscatter coefficient using a spherical Gaussian scatterer model (SGM)^30^. Finally, color-coded parametric maps for each estimated quantitative ultrasound parameter were constructed by generating a spatial map of the parameter values computed over all window blocks. The mean values of quantitative ultrasound parameters were determined by averaging QUS parametric map values. From the tumour core and 5-mm margin regions in QUS parametric images, two core-to-margin related parameters were calculated including core-to-margin ratio (CMR) and core-to-margin contrast ratio (CMCR)^24^. CMR compares the level of desired signal to the background noise, and CMCR is like CMR but also considers bias in an image.

### Texture analysis

A statistical texture analysis technique was applied on QUS parametric images based on the concept of a grey-level co-occurrence matrix (GLCM). The GLCM represents the statistical angular relationship between neighbouring pixels, as well as the distance between them^23^. Four texture features, including contrast (CON), correlation (COR), homogeneity (HOM), and energy (ENE), were determined based on the statistical information provided by GLCM analysis. QUS parametric maps of the MBF, SS, SI, ASD and AAC from core and margin regions underwent a GLCM-based texture analysis process to extract these four texture features. In texture analysis, the contrast feature represents location-dependent gray level variations in an image. The energy features measures textural uniformity while the homogeneity measures the incidence of pixels pairs of different intensities. As the frequency of pixel pairs with close intensities increases, homogeneity increases. The correlation feature measures the linear dependency among neighbouring pixels. In this study, a total of 40 textural features (four features for each of the five QUS parametric maps) were computed.

Texture-derivative analysis was subsequently applied to the QUS parametric images. In contrast to the previous texture analysis approach that produces averaged texture measures, texture-derivate analysis works by creating intermediary texture-encoded maps using a sliding window analysis with a 15-pixel by 15-pixel window. Each pixel in these maps represents a quantification of local textures across the window^18^. A second-pass GLCM based texture analysis was subsequently performed on these maps, resulting in texture-derivate features. In the end, a total of 201 features, including mean QUS, core-to-margin, texture, and texture-derivative features were extracted from each patient’s ultrasound RF data.

The diagram of QUS, core-to-margin, GLCM based texture, and texture-derivative parameter estimation from the ultrasound data are summarized in Supplementary Fig. 1.

### Classification and statistical analysis

**Figure 1:**
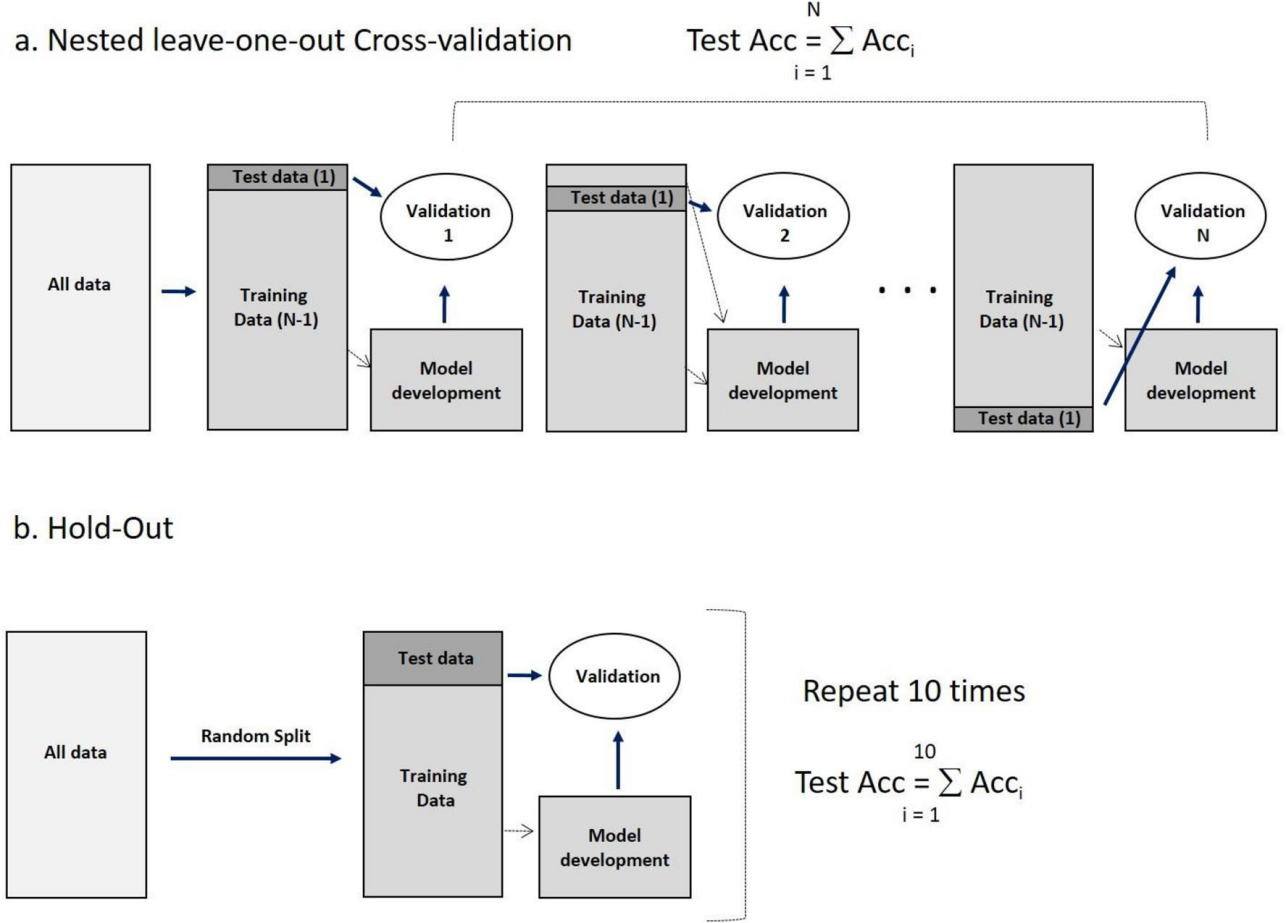
Validation method. (**a**) Nested leave-one-out cross-validation method. Nested cross-validation is performed in two layers to achieve training and validation separation. In the outer layer one sample was separated for validation and the rest of the data was used to develop a model. In the inner layer, the training data was used to develop classification model. Finally obtained model was tested with test data set. (**b**) Hold-out method. Here data set is divided into test and training data randomly. The training was used to develop classification model and tested with test data set. This process was repeated 10 times and average performance was calculated.

ANOVA followed by a Bonferroni multiple comparisons Tukey test was used to compare the extracted means of QUS parameters, texture features, texture-derivative features, and tumour molecular subtype between responders and non-responders. To develop a tumour response prediction model, a nested cross-validation was performed on the parameters determined from ultrasound data^31,32^. The nested cross-validation is performed in two layers to achieve training and validation separation. In this study, leave-one-out and holdout cross-validation were investigated. In leave-one-out nested cross-validation (Fig. 1a), in the outer layer, one sample was separated for validation, and the rest of the data was used to develop a model. In the internal layer, the remaining 207 samples were used for feature selection and classifier parameter tuning. A developed model was then validated with one left sample, which was split at the beginning. This process was repeated 208 times by leaving different one sample for validation and by using a different 207 samples to develop a new model from scratch. The overall performance was then calculated as a mean of classification performances of the 208 separately developed models on different one sample left for validation, which was not involved in developing the models. In hold-out cross-validation (Fig. 1b), 20% and 10% of the data was separated for validation, and 80% and 90% of the data, respectively, were used for model development. This process was repeated 10 times by randomly selecting 20%, and 10% of the data for validation and 80% and 90% for training process, respectively. Finally, overall performance was then calculated as a mean of classification performances of the 10 separately developed models. Classification metrics that include sensitivity, specificity, accuracy, positive predictive value (PPV), negative predictive value (PPV) and AUC were estimated to assess model performance.

In order to detect tumour response, a multi-feature response classification was performed using k-nearest neighbour (KNN) and support vector machine (SVM) with radial basis function (RBF) kernel^33^ methods. In KNN model development, the number of nearest-neighbor, k, was set to an odd number (k = 1, 3, and 5) to avoid tied classes in this binary class case. Finally, the optimal number of nearest neighbour was tuned to achieve the best classification performance. In SVM-RBF model development, two classifier parameters including, the penalty for misclassification (C) and the width of a radial basis function (γ) were tuned. Here, optimal values for these two parameters were selected by grid search with the range of C = 2^8^, 2^9^, 2^10^,…,2^15^ and γ = 2^–18^, 2^–17^, 2^–16^,…2^–5^.

The best feature subset was selected from training data set based on the maximal relevance minimal redundancy (mRMR) criterion^34^. This resulted in a reduced train dataset that consists of the 50 best mRMR features. From these best mRMR features, the optimal features were selected using the sequential-forward selection (SFS) method. To avoid the curse of dimensionality, the maximum number of features to select was set to 10 based on rule of thumb^35^. We implemented the synthetic minority over-sampling technique (SMOTE) to account for class imbalance^36^. Tumour response prediction model development was performed on this balanced reduced training dataset. The flow diagram of the training process, including feature selection, data balancing, and feature selection algorithm, is shown in Supplementary Fig. 2.

## Results

### Patient, tumour and treatment characteristics

The clinical and pathological characteristics of the patients involved in this study are summarized in Table 1. The average age of patients was 51 ± 11 years. The average tumour size along the longest axis before treatment was 5.0 ± 2.7 cm. Among patients, 89% had invasive ductal carcinoma and 4% had lobular carcinoma. Five percent (5%) of patients had grade I tumours, 38% had grade II tumours, and 49% had grade III tumours incorporating patients with locally advanced and earlier-stage breast cancer receiving neoadjuvant chemotherapy treatment. All of the patients included in this analysis had completed systemic therapy as planned. The most common chemotherapy regimen used was AC-T (Adriamycin, Cyclophosphamide, and Paclitaxel) in 59%, followed by FEC-D (5-Fluorouracil, Epirubicin, Cyclophosphamide, and Docetaxel) in 28%. Along with neoadjuvant chemotherapy, 31% of patients received trastuzumab in the neoadjuvant setting. Pathological complete response (pCR) was seen in 20% patients. The average tumour size along the longest axis after treatment was 2.7 ± 3.4 cm. According to response grading, 161 and 47 patients were detected as responders and non-responders to neo-adjuvant chemotherapy treatment, respectively. Tumours were grouped based on molecular subtype, including ERBB2 + (ER−, PR−, HER2+), triple negative (ER−, PR−, HER2−), Luminal-A (ER+ and/or PR+, HER2−), and Luminal-B (ER+ and/or PR+, HER2+). Among responding patients, 14%, 25%, 36%, and 25% were ERBB2+, triple negative, Luminal-A, and Luminal-B, respectively. Among non-responding patients, 0%, 26%, 64%, and 11% were ERBB2+, triple negative, Luminal-A, and Luminal-B, respectively. The 5-year recurrence-free survival (RFS) for the responders and non-responders was 82% and 65%, respectively, with a *p* value of 0.0002. The 5-years disease free survival rate calculated for ERBB2+ triple negative, Luminal-A, and Luminal-B molecular type breast cancer from the patient population were 85%, 76%, 81%, and 75%, respectively. They were not significantly different (*p* = 0.96). All patient characteristics, tumour properties, hormone receptor overexpression, and treatments administered details are presented in Supplementary Table 1.

**Table 1:** Clinical and pathologic characteristics of breast cancer patients receiving NAC.

Characteristics	R (N = 161)	NR (N = 47)	All (N = 208)
Age (year)	50 ± 12	52 ± 11	51 ± 11
Menopause			
Postmenopausal (%)	41	36	40
Premenopausal (%)	47	57	52
Perimenopausal (%)	12	7	8
Initial tumour size (cm)	5.2 ± 2.8	5.0 ± 2.6	5.0 ± 2.7
Histology			
IDC (%)	93	83	92
ILC (%)	3	9	4
IMC (%)	4	8	4
Group Stage			
Stage II (%)	50	61	52
Stage III (%)	48	37	46
Not reported (%)	2	2	2
Tumour Grade			
Grade I (%)	6	9	5
Grade II (%)	34	43	38
Grade III (%)	50	47	49
Not reported (%)	10	1	8
Molecular Subtype			
ERBB2 + (%)	14	0	11
Triple Negative (%)	25	26	25
LuminalA (%)	36	64	42
LuminalB (%)	25	11	22
Treatment			
ACT (%)	53	68	59
FECD (%)	30	23	28
Others (%)	17	9	13
Residual tumour size (cm)	1.8 ± 2.2	5.9 ± 4.5	2.7 ± 3.4

### Quantitative ultrasound, texture and texture-derivative parameters

**Figure 2:**
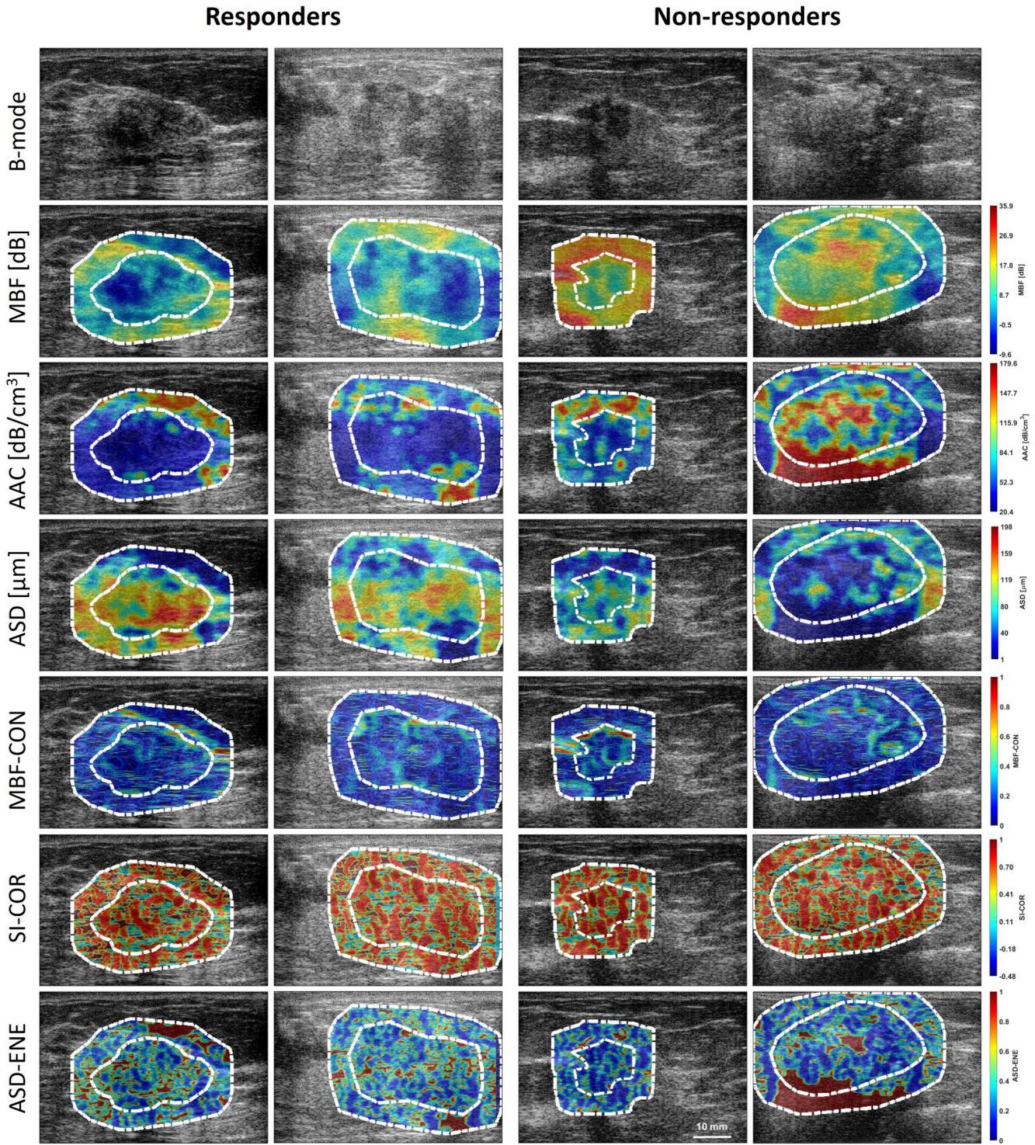
QUS and Texture images of response group. Representative B-mode, MBF, AAC, ASD, MBF-CON, SI-COR, and ASD-ENE parametric and texture images with tumour core and margin regions of two responders and non-responders. MBF: Mid-band fit; AAC: average acoustic concentration; ASD: average scatterer diameter; SI: spectral intercept; CON: Contrast; COR: correlation; ENE: energy.

Representative ultrasound B-mode, QUS parametric, and QUS-texture images corresponding to responding and non-responding patients, acquired prior to chemotherapy treatment, are displayed in Fig. 2. MBF and AAC parametric images demonstrated less tissue stiffness in both core and margin regions for responders compared to non-responders. ASD parametric images exhibited higher scatter diameters for responders than non-responders. A total of 201 features were determined from RF data acquired from breast cancer patients. Statistical analysis using unpaired *t* tests was performed to compare those QUS, core-to-margin, texture, and texture-derivative features between responders and non-responders acquired before treatment. Among the 201 features, 9 exhibited significant differences (*p* < 0.05) between responder and non-responder groups. The box plots for those features with significant differences, along with an overlaid scatterplot of the distribution of responding and non-responding patient values, are presented in Supplementary Fig. 3. The backscatter intensity parameters from the tumour core and margin regions, including *Core* MBF, *Margin* MBF, and *Core* AAC, were significantly higher in non-responders, as displayed in Fig. 2. The core-to-margin related parameters, including *CMR*-MBF, and *CMR*-AAC, were significantly lower and *CMCR*-AAC were significantly higher in responders, demonstrating a significantly difference in tissue stiffness between tumour core and margin region and less variation within the tumour core region in responder group, as visualized in Fig. 2. Texture-derivative parameters derived from scatter size parametric images from tumour core region, including *Core* ASD-ENE-HOM, *Core* ASD-ENE-CON, and *Core* ASD-ENE-ENE showed significant difference between two response groups, demonstrating the existence of orderly organized scatterers with broader varying sizes within tumour core region in responder group, as shown in Fig. 2.

### Classification performances

**Figure 3:**
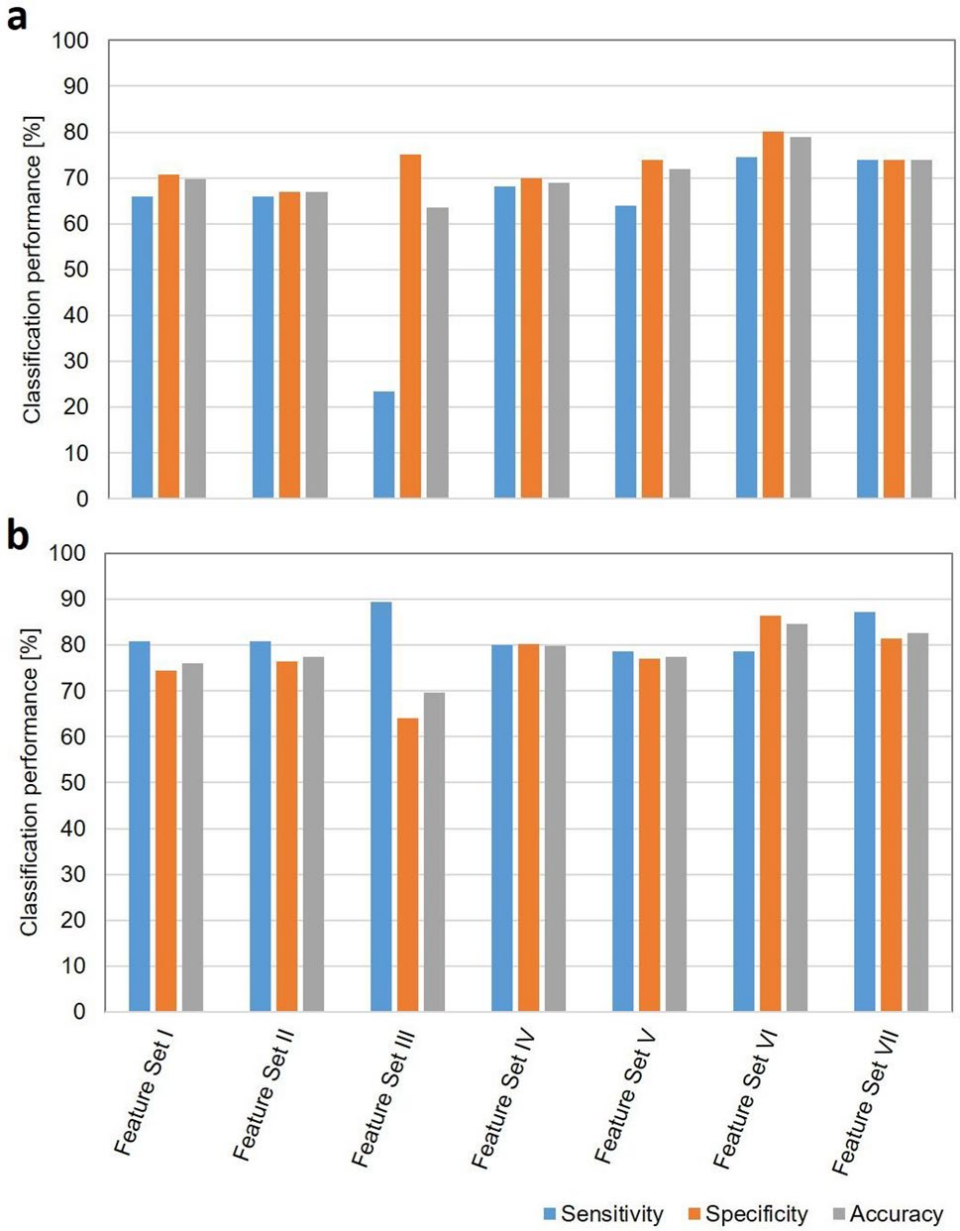
Summary of classification performance with leave-one-out cross-validation. Tumour response classification results obtained using (**a**) KNN and (**b**) SVM-RBF algorithms based on seven feature set types using leave-one-out cross-validation approach. The classification algorithms developed using both KNN and SVM-RBF classifiers exhibited the best performance with the feature set including QUS-texture-derivative parameter and tumour molecular subtype (Feature Set VI). Feature Set I: QUS + Texture + Core-to-Margin; Feature Set II: Texture Derivative; Feature Set III: Molecular Subtype; Feature Set IV: QUS + Texture + Core-to-Margin + Texture-Derivative; Feature Set V: QUS + Texture + Core-to-Margin + Molecular Subtype; Feature Set VI: Texture Derivative + Molecular Subtype; Feature Set VII: QUS + Texture + Core-to-Margin + Texture-Derivative + Molecular Subtype.

Two different classifiers were investigated with leave-one-out and hold-out cross-validation approaches, and their performances were compared. Figure 3 shows bar plots illustrating the classification performance of the models developed using KNN and SVM-RBF classifiers with the following feature sets: (I) features including mean QUS, texture and core-to-margin parameters from core and margin region, (II) features including texture-derivative parameters from core and margin, (III) features including tumour molecular subtype, (IV) features including mean QUS, texture, texture-derivative, core-to-margin parameters from core and margin region, (V) features including mean QUS, texture, core-to-margin parameters from core and margin region, and tumour molecular subtype, (VI) features including texture-derivative parameters from core and margin region, and tumour molecular subtype, and (VII) features including all features with leave-one-out cross-validation. The algorithms developed using KNN provided the best classification performance from the combination of feature sets. Table 2 presents the optimal features selected using KNN methodology. In the classification algorithm development using the KNN classifier, most features were selected from tumour core region. Combining tumour molecular subtype with the mean QUS and texture features increased the classification performance to an accuracy of 72%. The best performance obtained using KNN was from the feature set including texture-derivative parameters and molecular subtype (VI) with a sensitivity of 74%, specificity of 80%, accuracy of 79%, PPV of 52%, NPV of 91% and AUC of 0.76. In compareison to the KNN algorithm, the SVM-RBF classifier performed well in differentiating responder and non-responder groups from all type of feature sets. The best performances were obtained from three feature sets using an SVM-RBF classifier in differentiating responder and non-responder groups (feature sets IV, VI and VII). Table 3 displays optimal features selected from these feature sets during the training process in classification model development using SVM-RBF classifier. The parameters determined from ultrasound RF data, including mean QUS, core-to-margin, texture and texture-derivative parameters could alone differentiate the response groups with a sensitivity of 80%, specificity of 80%, accuracy of 80%, PPV of 54%, NPV of 93%and AUC of 0.83 using SVM-RBF (feature set IV). Combining molecular subtype information with these parameters slightly increased the classification performance to a sensitivity of 87%, specificity of 81%, accuracy of 83%, and AUC of 0.87 (feature set VII). Similar to the KNN model, the best performance using SVM-RBF was obtained from a feature set including texture-derivative parameters and molecular subtype (VI) with a sensitivity of 79%, specificity of 86%, accuracy of 85%, PPV of 63%, NPV of 93% and AUC of 0.87. The summary of the classification performance results using KNN and SVM-RBF classifier based on various type of feature set with leave-one-out cross-validation approach is presented in Supplementary Tables 2a and 2b, respectively.

**Table 2:** Optimal features selected for tumour response classification using KNN algorithm developed based on ultrasound data (Feature Set IV), ultrasound data + molecular subtype (Feature Set VII), and the best performed feature set (Feature Set VI) with leave-one-out cross validation approach.

No	Only ultrasound data (Feature Set IV)	Ultrasound data + Molecular subtype (Feature Set VII)	Best model feature set (Feature Set VI)
1	*Core* ASD	ERBB2 +	ERBB2 +
2	*Core* ASD-COR	Luminal-B	Luminal-B
3	*Core* ASD-ENE	*Core* ASD-HOM-COR	Luminal-A
4	*Core* MBF-HOM-HOM	Luminal-A	*Core* ASD-ENE-ENE
5	*Core* SS-COR-ENE	*Core* SS-HOM-ENE	*Core* ASD-HOM-COR
6	*CMR-*MBF	*Core* ASD-ENE	*Margin* ASD-ENE-ENE
7	*Core* SS-HOM-ENE	*Core* MBF	*Core* SS-HOM-ENE
8	*Core* AAC-HOM	*Margin* MBF-CON-COR	*Core* ASD-ENE-HOM
9	*Core* SI-CON	*Core* AAC-COR	*Core* SI-CON-COR
10	*CMR-*AAC	*Core* MBF-HOM-HOM	*Margin* MBF-CON-COR

Feature Set IV: QUS + Texture + Core-to-Margin + Texture-Derivative; Feature Set VI: Texture-Derivative + Molecular Subtype; Feature Set VII: QUS + Texture + Core-to-Margin + Texture-Derivative + Molecular Subtype.

**Table 3:** Optimal feature selected for tumour response classification using SVM-RBF algorithm developed based on ultrasound data (Feature Set IV), ultrasound data + molecular subtype (Feature Set VII), and the best performed feature set (Feature Set VI) with leave-one-out cross validation approach.

No	Only ultrasound data (Feature Set IV)	Ultrasound data + Molecular subtype (Feature Set VII)	Best model feature set (Feature Set VI)
1	*Core* MBF-ENE-ENE	Luminal-A	Luminal-A
2	*CMCR*-SI	*Core* AAC-COR	*Core* SI-COR-HOM
3	*CMCR*-AAC	*CMCR*-AAC	*Margin* AAC-CON-COR
4	*CMCR*-ASD	*Core* SI-COR-HOM	*Core* MBF-CON-COR
5	*Core* SS-COR-ENE	*CMCR*-SI	*Core* SI-COR-ENE
6	*Margin* AAC-COR-HOM'	*Core* SI-COR	*Margin* AAC-COR-CON
7	*CMR*-SI	*Core* MBF-ENE	*Margin* MBF-CON-COR
8	*Core* MBF-ENE-COR	*Core* SI-COR-ENE	*Core* SS-COR-CON
9	*Margin* MBF	*Core* MBF-CON-COR	*Core* MBF-ENE-HOM
10	*Margin* MBF-CON-COR	*CMCR*-ASD	*Margin* AAC-COR-HOM

Feature Set IV: QUS + Texture + Core-to-Margin + Texture-Derivative; Feature Set VI: Texture-Derivative + Molecular Subtype; Feature Set VII: QUS + Texture + Core-to-Margin + Texture-Derivative + Molecular Subtype.

**Figure 4:**
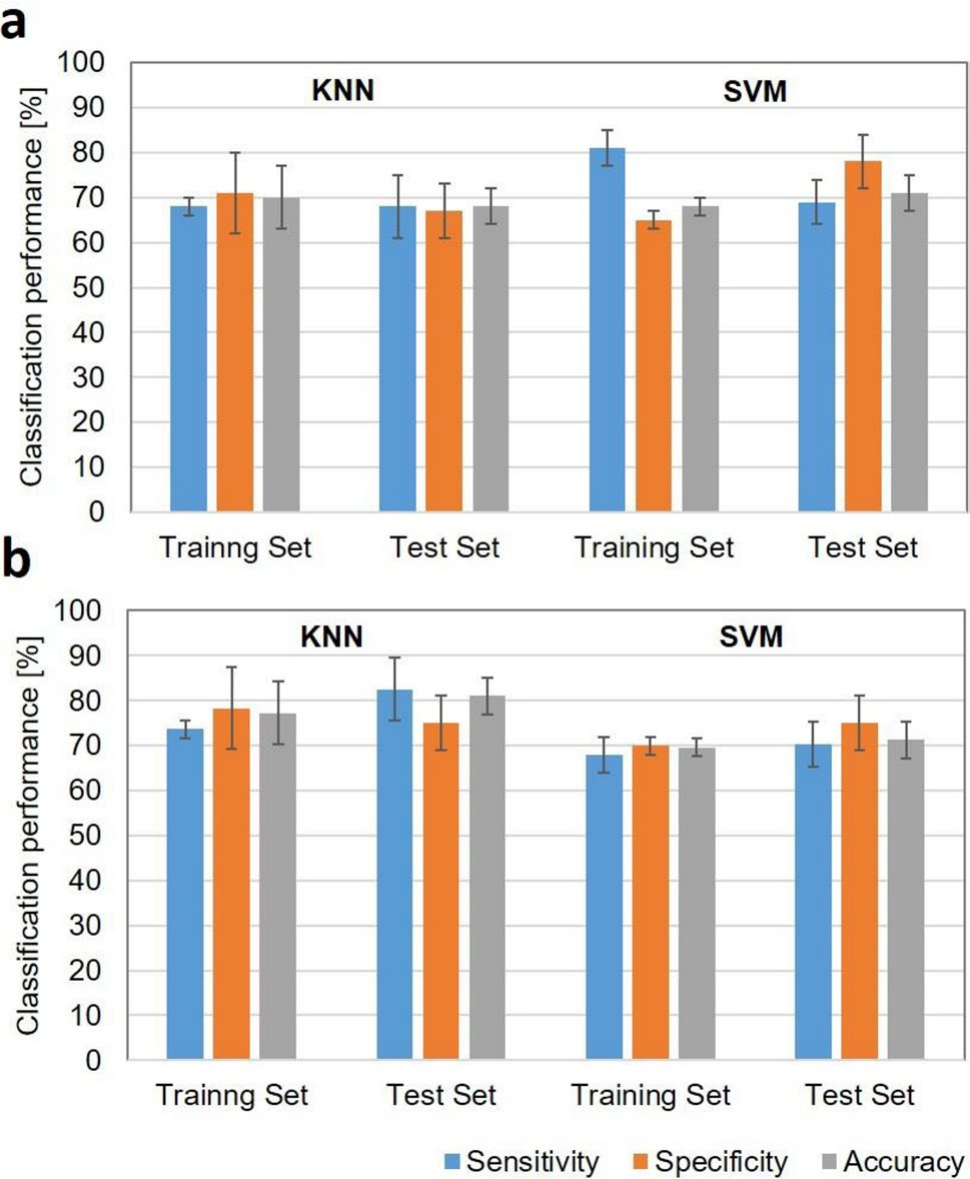
Summary of classification performance with hold-out validation. Average tumour response classification results obtained from 10 hold-out data set using KNN and SVM-RBF algorithms. The top panel (**a**) shows data with 20% testing (80% training) and the bottom plot (**b**) with 10% testing (90% training) hold-out approaches. The classification performance with 90% training was approximately similar to those with leave-one-out approach with bias and variance errors of 21% and 3%, respectively*.*

Figure 4 displays the average performance on the training and test data sets using mean QUS, core-to-margin, texture, texture-derivative and molecular subtype with 10 hold-out validations, employing KNN and SVM-RBF algorithms for holding out 20% of data, and 10% of data. In a hold-out cross-validation approach, the model developed using KNN and SVM-RBF classifier exhibited similar performance in differentiating response groups.

For the 20% hold-out cross-validation, the classification accuracy and AUC for the KNN algorithm were 70 ± 7% and 0.71 ± 0.07, respectively, from the training set, and 68 ± 4% and 0.69 ± 0.02% from the test set. The bias and variance errors for this model were 30% and 2%, respectively. Similarly, the classification accuracy and AUC for the SVM-RBF algorithm were 68 ± 2% and 0.73 ± 0.04 from training set, 71 ± 4% and 0.71 ± 0.04% from independent test set, respectively. The bias and variance errors for this model were 32% and 3%, respectively.

For the 10% hold-out cross-validation, the classification accuracy and AUC for the KNN algorithm were 77 ± 2.5% and 0.76 ± 0.05from the training set, 81 ± 2.2% and 0.80 ± 0.03% from the test set, respectively. The bias and variance errors for this model were 21% and 3%, respectively. Similarly, the classification accuracy and AUC for the SVM-RBF algorithm were 70 ± 0.6% and 0.72 ± 0.03 from the training set, 71 ± 2.5% and 0.71 ± 0.07% from independent test set, respectively. The bias and variance errors for this model were 30% and 2%, respectively. The summary of classification performance results with 20% (80% training) and 10% (90% training) testing hold-out approach is presented in Supplementary Table 3a and 3b, respectively.

## Discussion

This study demonstrated, for the first time, the potential of combining quantitative ultrasound, texture, texture derivative, and molecular subtype analysis techniques to predict cancer treatment responder and non-responder among breast cancer patients before starting neo-adjuvant chemotherapy. All patients underwent a core needle biopsy to confirm a cancer diagnosis and to determine tumour histological subtype and molecular subtype before treatment. A total of 201 features were determined from ultrasound data acquired from patients, including 5 QUS parameters from tumour core and tumour margin, 20 texture parameters from core and margin, 80 texture-derivative parameters from core and margin, 10 core-to-margin parameters, and tumour attenuation before treatment.

To understand the relationship of QUS, texture, and texture-derivative parameters determined from ultrasound-RF data with treatment response, we investigated the correlation between these features and patient tumour response. Among all QUS parameters, the backscatter intensity-related parameters from tumour core and margin regions, as well as ore-to-margin parameters, revealed significant differences between response groups. Additionally, specific texture-derivative features from the tumour core scatterer size—energy feature parametric images exhibited significant differences. Generally, backscatter intensity-related parameters, including the MBF and AAC, are strongly related to scatter number density and elastic properties^15,30,37^. In this study, the range of scatter size determined from ultrasound data acquired in both tumour and margin region was approximately 80–182 µm, representing lobule diameters observed in histopathological images. These results suggest that these two different type of responding group have different lobular number density in both tumour core and surrounding regions and different uniformity in size distribution in core region. These finding are reflected in the MBF and AAC parametric images constructed from responder data, demonstrating less tissue stiffness in both tumour core and margin regions compared to non-responder group (Fig. 2). Significant differences in backscatter parameters estimated from the margin region, as well as parametric image texture and texture-derivative parameters between two response groups, reveal the existence of microscopic infiltration from the tumour core to the surrounding region, particularly in non-responding patients. This is visualized in MBF and AAC images by significantly different values between core and margin region in responder group and no different in the non-responder group. Among molecular subtypes, majority (64%) of Luminal-A type tumours were in non-responding group, and none of the ERBB2+ type tumours were in non-responding group. Previous studies have demonstrated that molecular subtype is a powerful independent predictor of chemotherapy response rate and overall survival. There were reported significant difference in response rates and overall survival of breast cancer patients with different molecular subtypes, including HER2+, triple negative, and ER and/or PR+ with HER2- status^27^. Another study reported a lower pCR rate in Luminal-A and higher in Her2+ disease for neoadjuvant chemotherapy^38^. This was reflected in our study population. However, the calculated survival rate of these four molecular subtype tumour did not reveal the significant difference between these types. This is due to lack of sufficient sample in each molecule subtypes from our breast cancer patient population. Nevertheless, compared to responder group, number of Luminal A type tumour was higher and Her2+ type tumour was lower (12%) in our non-responder population.

**Figure 5:**
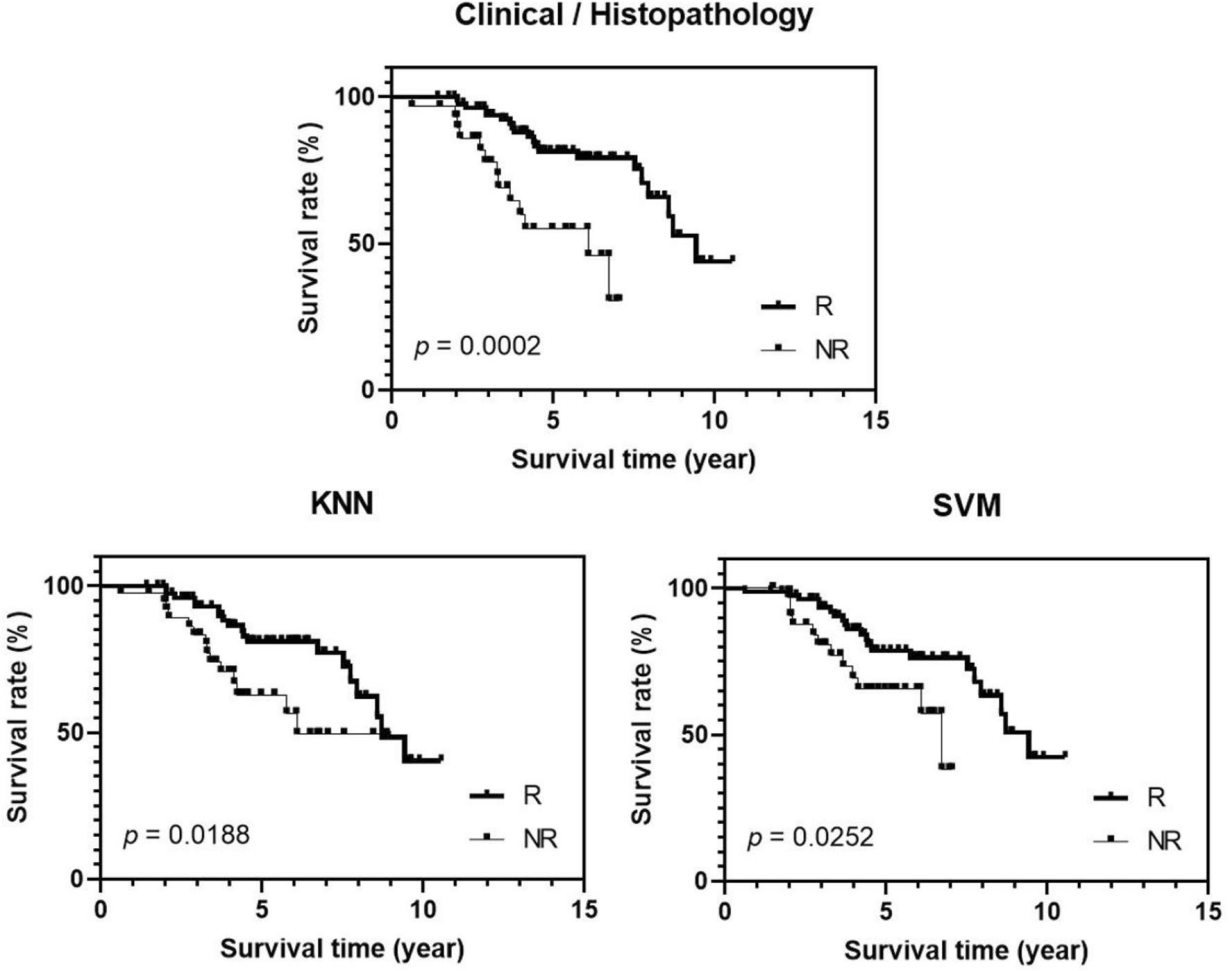
Recurrence free survival curves for neo-adjuvant chemotherapy treatment responders and non-responders. Patients were differentiated based on clinical/pathology after treatment, and also based on baseline texture-derivative parameters combined with molecular subtype using the KNN and SVM-RBF algorithm.

In this study, multi-feature classification analyses were conducted on different feature sets, including mean QUS & texture from core and margin, texture-derivative parameters from core and margin, molecular subtype, and combination of these feature sets. Models developed from mean QUS & texture, and texture-derivative parameter exhibited similar performances with accuracies of approximately 69% and 70% using KNN and SVM-RBF, respectively. These performances were improved by including molecular subtype. The best performances were achieved from the feature set including texture-derivative parameters and molecular subtype, with accuracies of 79% and 85% using KNN and SVM-RBF, respectively. Comparing the performance of algorithms using two different classifiers, the better performances were obtained with the model developed using an SVM-RBF classifier based on all feature sets. Mostly, core-to-margin parameters that include core and margin region are selected (Table 3). This confirms the result presented in our previous finding where it was reported that the existence of microscopic extension in tumour surrounding regions affect the tumour response to NAC. The best classification performance was obtained by the combination of texture-derivative parameters from tumour core and margin, and molecular subtype, particularly texture-derivative parameters from backscatter intensity-related parametric maps, and Luminal-A type, with a sensitivity of 79%, specificity of 86%, accuracy of 85%, PPV of 63%, NPV of 93%, and AUC of 0.87. This agrees with a previous finding that response rate is significantly lower for Luminal-A type tumours compared to other molecular subtypes, and molecular subtype is one of the key predictor for treatment response. Combining molecular subtype with intra-tumoural heterogeneity reflected by texture-derivative parameters could easily predict the response type before treatment. The PPV and NPV values reveal that the our model has 63% chance in predicting non-responding patient and 93% chance in predicting responding patient accurately. Even though the change of predicting non-responder is moderate, there is a higher change of predicting responder. This finding reveals that, based on our tumour response model performance, treatment for the responding patient will not be changed from standard neoadjuvant treatment procedure. The lower value of PPV is due limited number of non-responding patient, which means the prevalence value is 22% in our patient population. Several previous studies reported that pathological response is a prognostic indicator for long-term, disease-free and overall survival^39^. This was confirmed in this current study. As expected, clinical outcomes were significantly different between the responders and non-responders, as defined by the modified response grading system criteria and demonstrated in survival plots (Fig. 5). The obtained models could differentiate two response-type patients’ outcomes with good agreement with those based on histopathology and clinical outcomes determined after surgery. This result implies that the proposed classification models, based on combined quantitative ultrasound-texture biomarkers and molecular subtype as early survival-linked surrogates of response to cancer-targeting therapies, could facilitate switching from an inefficient treatment regimen to a more effective one on an individual patient-basis before starting treatment.

For the generalization of the proposed tumour response prediction models using KNN and SVM-RBF classifiers, we investigated their performance with a hold-out cross-validation approach. The classification performance with 20% testing and 80% training hold-out was, as expected, lower than result obtained with leave-one-out cross-validation approach, with bias and variance errors approximately at 30% and 2% respectively. However, the performance using KNN classifier with 10% testing and 90% training hold-out was approximately similar to that with leave-one-out approach, with bias and variance errors approximately at 21% and 3% respectively*.* The range of bias and variance errors from 10 repeated 20% testing hold-out validation was 31 to 35% and 1 to 11%, respectively. For 10 repeated 10% testing hold-out validations, the range was15 to 25% and 0.3 to 9%, respectively. This high variance and bias with 20% testing holdout reveal that the number of sample in training process is not sufficient enough to perform an adequately powered hold-out validation. This suggest that for our current patient population size, leave-one-out is the better cross-validation choice to investigate the classification performance of the response group based on combined quantitative ultrasound, texture-derivative and molecule subtype analyses.

In a previous study investigating tumour response prediction with a population of 56 patient based on mean QUS, texture, and core-to-margin parameters from core and margin regions, a classification performance with accuracy of 88% was reported^24^. In another study involving 100 patient, tumour response prediction was performed based solely on mean QUS, texture, and texture-derivative parameters from tumour core region, reporting a performance accuracy of 82%^18^. However this study showed slightly lower performance with those feature sets. This discrepancy is attributed to the increase variety of tissue microstructures in training sample population resulting from the inclusion of more patients in the model development. By combing tumour molecular subtype with mean QUS, texture and texture-derivative parameters, a significant improvement in the performance of tumour response prediction could be achieved.

In conclusion, this work demonstrates that combining texture analysis of quantitative ultrasound features with molecular subtype can accurately detect tumour response before neoadjuvant chemotherapy using a machine learning approach. While the current population appears reasonably good for tumour response prediction with leave-one-out cross-validation approach, a larger cohort of patients in the future should improve the generalizability and robustness of the prediction, even using hold-out validation approach. Nevertheless, this study shows that molecular and QUS-texture markers can serve as a prior treatment survival-linked surrogate of response to cancer-targeting therapies—leading the way towards personalized medicine and facilitating the selection of an appropriate treatment regimen on an individual patient basis.

### Supplementary Information

Supplementary Information.

## Data Availability

Data collected and analyzed in this study are available from the Sunnybrook Research Institute Research Ethics Board approved study “Pilot Investigation of Ultrasound Imaging and Spectroscopy and Ultrasound Imaging of Vascular Blood Flow as Early Indicators of Locally Advanced Breast Cancer Response to Neoadjuvant Treatment”. Since this is patient data, the authors are legally bound to keep it confidential. Data can be made available upon request and review by Institutional Review Board (IRB). Data requests may be sent to Dr. Kullervo Hynynen, Vice-president, Research & Innovation, Sunnybrook Research Institute (khynynen@sri.utoronto.ca).
