# Supplementary material for: A priori prediction of breast cancer response to neoadjuvant chemotherapy using quantitative ultrasound, texture derivative and molecular subtype
Source: Sci Rep. 2023 Dec 19;13:22687. doi: 10.1038/s41598-023-49478-3 (PMC10730572; doi:10.1038/s41598-023-49478-3)
Supplement: Supplementary file 1 — Supplementary Information. [file 41598_2023_49478_MOESM1_ESM.docx]

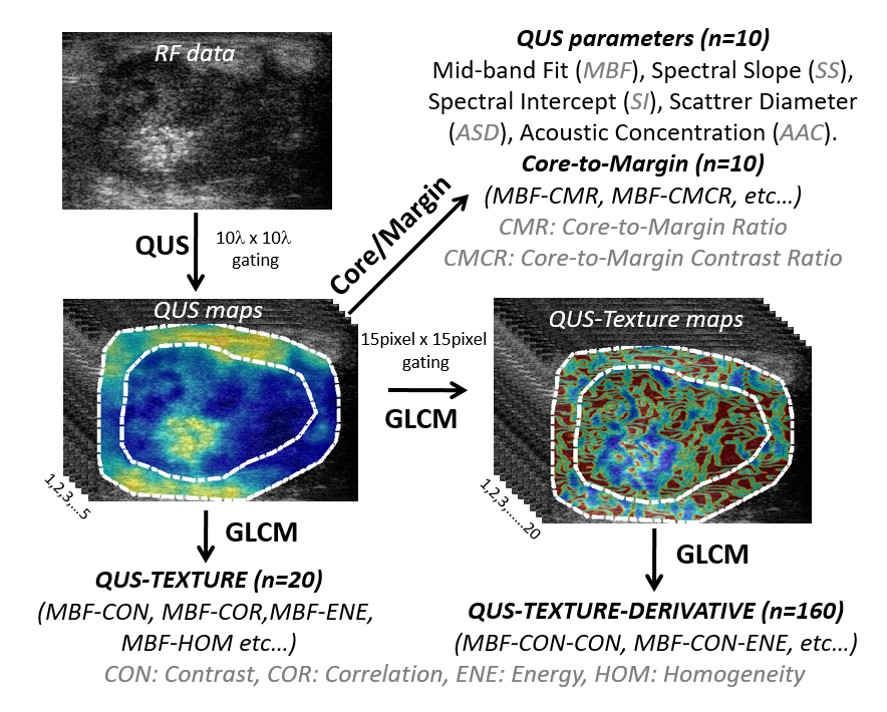


**Supplementary Figure 1.** **Ultrasound RF data processing.** The QUS, GLCM based texture and texture-derivative parameter estimation from the ultrasound data are presented. QUS: Quantitative Ultrasound technique; GLCM: grey-level co-occurrence matrix method. QUS maps: MBF, SS, SI, ASD, and AAC parametric maps (5 QUS maps). QUS –Texture maps: MBF-CON, MBF-COR, MBF-EN, MBF-HOM texture maps etc…. (20 QUS-Texture maps).


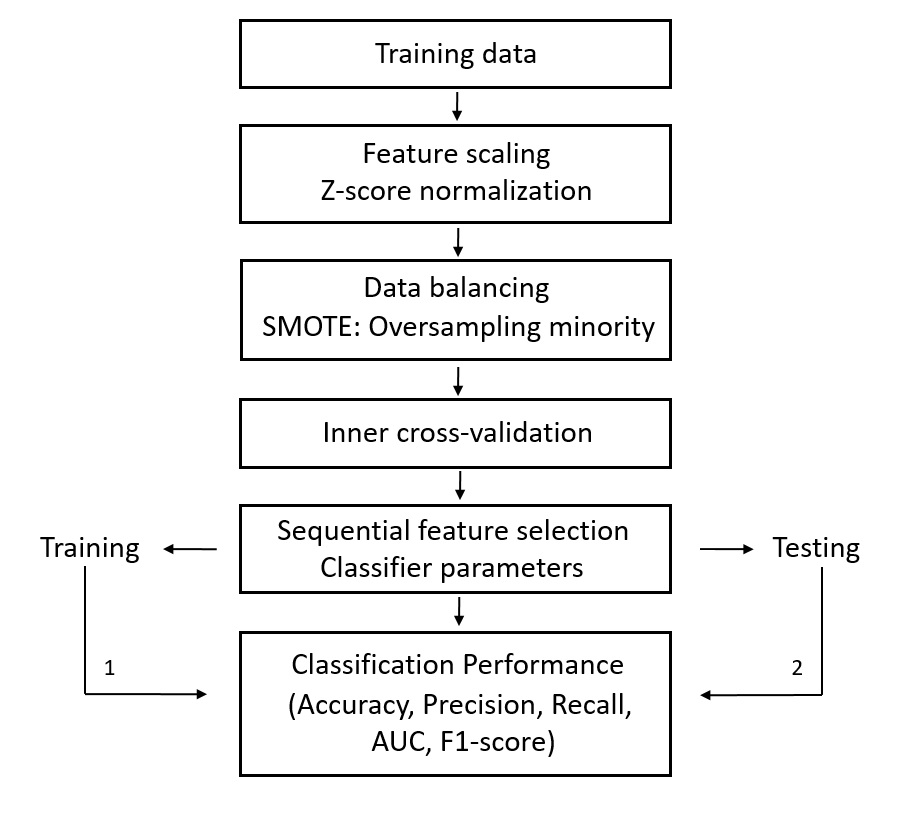


**Supplementary Figure 2.** The flow diagram of the training process including feature selection, data balancing and feature selection algorithm.


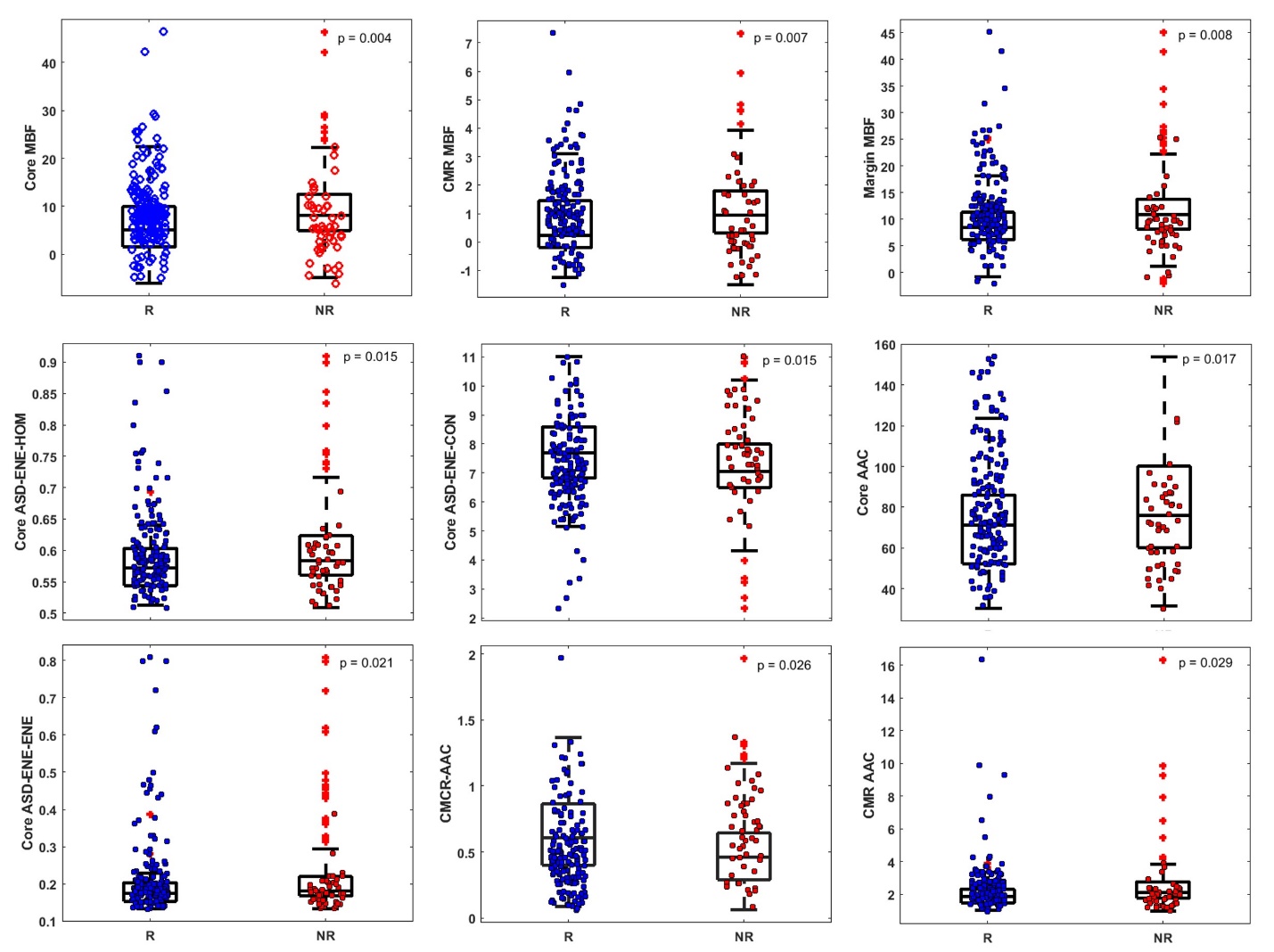


**Supplementary Figure 3.** Box and scattering plots of features that exhibited statistical significant difference (*p* < 0.05) between responder and non-responder groups among 201 features extracted from ultrasound data and are arranged in the order of *p* values. The bottom and top edges of the box represent the 25th and 75th percentiles, respectively. The central mark in each box indicates the median. The whiskers represent 1.5 times the interquartile range.

**Supplementary Table 1: Patient characteristics and neoadjuvant treatment response results.**

| **No** | **Age** | **Menopausal status** | **Pre-Tx tumour size (cm)** | **Histology** | **Tumour Grade** | **Group Stage** | **ER/**  **PR/**  **Her2** | **Treatment** | **Post-Tx tumour size** | **Response** |
| --- | --- | --- | --- | --- | --- | --- | --- | --- | --- | --- |
| 1 | 53 | post | 5.4 | IDC | I | IIIB | --+ | FECD + TRA | 0.0 | R |
| 2 | 53 | pre | 7.3 | IDC | I | IIIA | ++- | DE | 7.0 | R |
| 3 | 39 | pre | 5.3 | IDC | II | IIIA | +++ | DCB+TRA | 2.7 | R |
| 4 | 50 | pre | 4.6 | IDC | III | IIIB | +++ | ACT+TRA | 0.0 | R |
| 5 | 31 | pre | 5.0 | IDC | I | IIB | ++- | ACT | 1.4 | R |
| 6 | 46 | pre | 8.0 | IDC | III | IIA | --- | ACT | 6.4 | NR |
| 7 | 39 | pre | 10.0 | IDC | II | IIIA | +++- | FECD | 8.0 | R |
| 8 | 43 | post | 8.0 | IDC | II | IIIA | +++ | ACT+TRA | 0.0 | R |
| 9 | 48 | pre | 4.9 | IDC | III | IIIA | ++- | ACT | 1.4 | R |
| 10 | 36 | pre | 5.8 | IDC | III | IIIB | +++ | ACT | 11.4 | NR |
| 11 | 40 | post | 4.4 | IDC | III | IIIC | --- | ACT | 0.0 | R |
| 12 | 62 | post | 10.0 | IDC | III | IIA | --- | D+XRT | 0.0 | R |
| 13 | 36 | post | 8.0 | IDC | III | IIIA | +++ | ACT+TRA | 0.0 | R |
| 14 | 59 | post | 6.0 | IDC | III | IIB | --+ | ACT | 2.6 | R |
| 15 | 65 | pre | 6.7 | IDC | II | IIIA | +-- | ACT+TRA | 5.5 | NR |
| 16 | 38 | pre | 9.2 | IDC | II | IIIA | ++- | ACT | 4.5 | R |
| 17 | 53 | pre | 11.7 | IMC | III | N/A | --- | ACT | 9.4 | NR |
| 18 | 48 | pre | 9.0 | IDC | II | IIIA | +++ | ACT | 5.0 | R |
| 19 | 50 | pre | 5.3 | IDC | III | IIIA | --- | ACT | 4.0 | R |
| 20 | 49 | peri | 12.0 | IDC | III | IIIA | --+ | D+TRA | 0.0 | R |
| 21 | 46 | pre | 7.0 | IDC | III | IIIA | --- | ACT | 0.0 | R |
| 22 | 40 | post | 3.0 | IDC | III | IIB | -++ | ACT+TRA | 0.0 | R |
| 23 | 56 | pre | 3.2 | IDC | II | IIB | -++ | ACT+TRA | 0.2 | R |
| 24 | 49 | pre | 5.6 | IDC | II | IIIA | --+ | ACT+TRA | 0.1 | R |

**Supplementary Table 1 continue…**

| 25 | 47 | | post | | 5.2 | | IDC | | II | | IIB | | ++- | | FECD | | 6.5 | | R | |
| --- | --- | --- | --- | --- | --- | --- | --- | --- | --- | --- | --- | --- | --- | --- | --- | --- | --- | --- | --- | --- |
| 26 | 52 | pre | | 4.1 | | IDC | | N/A | | IIB | | ++- | | ACT | | 0.0 | | R | |  |
| 27 | 44 | | pre | | 9.9 | | IDC | | III | | IIIA | | +++ | | ACT+TRA | | 2.0 | | R | |
| 28 | 38 | | post | | 9.0 | | IDC | | II | | IIIB | | ++- | | ACT | | 2.9 | | R | |
| 29 | 58 | | pre | | 1.9 | | IDC | | III | | IIB | | --- | | ACT | | 0.0 | | R | |
| 30 | 36 | | pre | | 12.0 | | IDC | | I | | IIIB | | ++- | | ACT | | 8.0 | | R | |
| 31 | 38 | | pre | | 8.0 | | IDC | | III | | IIA | | --+ | | ACT | | 0.0 | | R | |
| 32 | 47 | | post | | 9.9 | | IDC | | II | | IIIB | | ++- | | ACT | | 18.0 | | NR | |
| 33 | 57 | | post | | 5.5 | | IDC | | III | | IIIA | | --- | | ACT | | 0.0 | | R | |
| 34 | 59 | | peri | | 4.7 | | IDC | | N/A | | IIIA | | ++- | | ACT | | 2.1 | | R | |
| 35 | 47 | | pre | | 7.4 | | IDC | | N/A | | IIIA | | --+ | | ACT+TRA | | 0.0 | | R | |
| 36 | 55 | | pre | | 12.8 | | IDC | | II | | IIIA | | ++- | | ACT | | 17.0 | | NR | |
| 37 | 62 | | post | | 10.0 | | IDC | | III | | N/A | | +++ | | FECD | | 7.0 | | R | |
| 38 | 32 | | pre | | 7.0 | | IMC | | N/A | | IIIA | | +++ | | ACT+TRA | | 7.4 | | R | |
| 39 | 38 | | pre | | 2.5 | | IDC | | III | | IIA | | --- | | ACT | | 3.8 | | NR | |
| 40 | 45 | | pre | | 6.0 | | IDC | | I | | IIIA | | +++ | | ACT+TRA | | 4.8 | | NR | |
| 41 | 55 | | post | | 10.5 | | IDC | | III | | IIIA | | --- | | ACT | | 0.1 | | R | |
| 42 | 59 | | post | | 8.0 | | IDC | | II | | IIIA | | +-+ | | FECD+TRA | | 0.0 | | R | |
| 43 | 45 | | pre | | 3.8 | | IDC | | II | | IIB | | ++- | | ACT | | 0.5 | | R | |
| 44 | 37 | | pre | | 3.6 | | IDC | | III | | IIB | | ++- | | ACTA | | 2.2 | | R | |
| 45 | 50 | | pre | | 9.0 | | IDC | | II | | IIIB | | +++ | | ACT+TRA | | 1.2 | | R | |
| 46 | 54 | | peri | | 3.6 | | IDC | | N/A | | IIB | | ++- | | DC | | 1.7 | | R | |
| 47 | 55 | | pre | | 1.6 | | IMC | | I | | IIB | | +-- | | DC | | 1.2 | | NR | |
| 48 | 50 | | post | | 7.3 | | IDC | | III | | IIIA | | --- | | FECD | | 2.1 | | R | |
| 49 | 55 | | post | | 3.4 | | IDC | | III | | N/A | | --- | | ACT | | 1.8 | | R | |
| 50 | 32 | | post | | 2.7 | | IDC | | II | | IIB | | +-+ | | ACT+TRA | | 0.1 | | R | |
| 51 | 64 | | post | | 8.7 | | ILC | | II | | IIIC | | ++- | | FECD | | 19.0 | | NR | |
| 52 | 67 | | post | | 2.5 | | IDC | | II | | IIA | | --- | | FECD | | 3.2 | | R | |
| 53 | 52 | | post | | 2.6 | | IDC | | II | | IIA | | --- | | FECD | | 2.5 | | R | |

**Supplementary Table 1 continue…**

| 54 | 56 | pre | 7.0 | IDC | II | IIIA | +++ | ACT+TRA | 8.4 | NR |
| --- | --- | --- | --- | --- | --- | --- | --- | --- | --- | --- |
| 55 | 45 | post | 2.3 | IDC | N/A | IIIB | +++ | FECD+TRA | 0.0 | R |
| 56 | 59 | post | 4.9 | IDC | II | IIA | ++- | FECD | 2.8 | NR |
| 57 | 67 | pre | 7.4 | IDC | III | IIIA | ++- | FECD | 3.3 | R |
| 58 | 49 | pre | 2.1 | IDC | II | IIA | +-+ | ACT+TRA | 0.0 | R |
| 59 | 62 | pre | 6.3 | IDC | II | IIB | --- | ACT | 12.6 | NR |
| 60 | 58 | post | 5.2 | IDC | I | IIB | +++ | ACT+TRA | 3.4 | R |
| 61 | 58 | pre | 4.0 | IMC | III | IIB | --+ | DCB+TRA | 0.0 | R |
| 62 | 45 | pre | 4.0 | IDC | II | IIB | ++- | ACT | 3.0 | NR |
| 63 | 29 | pre | 4.2 | IDC | III | IIB | ++- | ACT | 4.0 | NR |
| 64 | 79 | post | 3.9 | IDC | II | IIB | --+ | ACT+TRA | 0.1 | R |
| 65 | 42 | pre | 9.6 | IDC | N/A | IIIA | ++- | FECD | 3.0 | R |
| 66 | 66 | post | 3.0 | IDC | III | IIB | +-+ | FECD+TRA | 2.4 | NR |
| 67 | 38 | pre | 5.0 | IDC | III | IIB | --- | ACT | 5.0 | NR |
| 68 | 40 | pre | 11.7 | IDC | III | IIIA | +++ | ACT+TRA | 1.3 | R |
| 69 | 53 | post | 8.8 | IDC | II | IIIB | --- | FECD | 2.5 | R |
| 70 | 47 | pre | 3.5 | IDC | II | IIA | ++- | ACT | 4.0 | NR |
| 71 | 57 | peri | 3.9 | IDC | II | IIA | +-- | ACT | 3.3 | NR |
| 72 | 54 | post | 5.0 | IDC | II | IIB | ++- | ACT | 0.0 | R |
| 73 | 63 | post | 3.0 | IDC | II | IIA | ++- | ACT | 0.5 | R |
| 74 | 31 | pre | 9.5 | IDC | II | IIIA | ++- | ACT | 0.6 | R |
| 75 | 47 | peri | 2.4 | IDC | III | IIIC | --- | ACT | 0.0 | R |
| 76 | 41 | pre | 7.9 | IDC | II | IIIA | --+ | ACT+TRA | 0.2 | R |
| 77 | 43 | pre | 6.6 | IDC | II | IIB | ++- | ACT | 4.0 | R |
| 78 | 38 | pre | 4.8 | IDC | III | IIB | --- | ACT | 0.0 | R |
| 79 | 69 | post | 4.3 | IDC | I | IIB | ++- | FECD | 2.0 | R |
| 80 | 51 | post | 4.2 | IDC | III | IIB | --+ | ACT+TRA | 0.0 | R |
| 81 | 53 | post | 5.6 | IDC | III | IIIA | --+ | ACT+TRA | 0.2 | R |
| 82 | 55 | post | 7.9 | IDC | II | IIIA | ++- | ACT | 12.6 | R |

**Supplementary Table 1 continue…**

| 83 | 51 | pre | 2.2 | IDC | N/A | IIB | ++- | ACT | 0.5 | R |
| --- | --- | --- | --- | --- | --- | --- | --- | --- | --- | --- |
| 84 | 55 | post | 3.1 | IDC | III | IIB | +++ | FECD+TRA | 0.0 | R |
| 85 | 31 | pre | 4.0 | IDC | N/A | IIB | +-- | ACT | 1.7 | R |
| 86 | 41 | pre | 2.2 | ILC | III | IIB | ++- | ACT | 1.1 | R |
| 87 | 53 | Peri | 2.3 | IDC | II | IIB | ++- | FECD | 0.0 | R |
| 88 | 42 | pre | 3.1 | IDC | N/A | IIB | --- | ACT | 0.0 | R |
| 89 | 31 | pre | 4.0 | IDC | III | IIIA | --- | ACT | 0.0 | R |
| 90 | 32 | pre | 5.6 | IDC | III | IIIA | --- | ACT | 2.7 | R |
| 91 | 45 | pre | 8.1 | IDC | II | IIB | --+ | TC+TRA | 0.1 | R |
| 92 | 53 | pre | 5.3 | IDC | III | IIIA | --- | FECD | 0.0 | R |
| 93 | 58 | post | 5.3 | IDC | III | IIIA | +++ | FECD+TRA | 1.5 | R |
| 94 | 38 | pre | 10.8 | IDC | II | IIB | ++- | FECD | 4.9 | R |
| 95 | 72 | post | 3.3 | IDC | II | IIA | +-- | ACT | 0.2 | R |
| 96 | 41 | pre | 4.5 | IDC | III | IIB | --- | ACT | 2.0 | R |
| 97 | 48 | pre | 5.6 | ILC | II | IIB | ++- | FECD | 11.0 | NR |
| 98 | 83 | post | 7.7 | IDC | III | IIIA | +-+ | ACT+TRA | 0.0 | R |
| 99 | 51 | post | 4.5 | IDC | III | IIB | ++- | ACT | 3.5 | NR |
| 100 | 43 | pre | 9.0 | IDC | III | IIB | --- | ACT | 3.9 | R |
| 101 | 42 | pre | 5.0 | IMC | III | IIB | ++- | FECD | 8.0 | NR |
| 102 | 60 | post | 7.2 | IDC | III | IIB | +++ | ACT+TRA | 1.0 | R |
| 103 | 42 | pre | 6.3 | IDC | II | IIB | ++- | FECD | 8.4 | NR |
| 104 | 42 | pre | 4.1 | IDC | I | IIIB | ++- | ACT | 4.5 | NR |
| 105 | 47 | pre | 10.4 | IDC | II | IIIA | ++- | ACT | 4.5 | R |
| 106 | 45 | pre | 7.3 | IDC | II | N/A | ++- | FECD | 1.4 | R |
| 107 | 43 | pre | 8.5 | IMC | II | IIIA | --- | FECD | 0.01 | R |
| 108 | 45 | pre | 6.2 | IDC | II | IIIA | +++ | FECD+TRA | 4.0 | R |
| 109 | 50 | pre | 4.2 | IDC | II | IIB | +++ | FECD+TRA | 1.9 | R |
| 110 | 62 | post | 2.1 | IDC | I | IIA | --- | DCB | 0.0 | R |
| 111 | 69 | post | 7.3 | ILC | II | IIB | +++ | ACT+TRA | 0.0 | R |

**Supplementary Table 1 continue…**

| 112 | 42 | pre | 6.0 | IDC | II | IIA | +++ | FECD+TRA | 2.5 | R |
| --- | --- | --- | --- | --- | --- | --- | --- | --- | --- | --- |
| 113 | 72 | pre | 1.3 | IDC | II | IIA | +++ | TC+TRA | 0.0 | R |
| 114 | 70 | post | 5.8 | IDC | I | IIB | ++- | FECD | 14.4 | NR |
| 115 | 45 | pre | 2.0 | IDC | I | IIIC | ++- | FECD | 0.2 | R |
| 116 | 42 | pre | 3.8 | IDC | III | IIB | ++- | ACT | 1.0 | R |
| 117 | 63 | post | 8.6 | IDC | II | IIIA | ++- | ACT | 7.5 | NR |
| 118 | 52 | pre | 3.1 | IDC | II | IIB | --- | FECD | 0.2 | R |
| 119 | 61 | post | 8.2 | IDC | II | IIIC | ++- | ACT | 3.0 | NR |
| 120 | 54 | post | 2.3 | IDC | III | IIA | --- | FECD | 0.0 | R |
| 121 | 68 | post | 2.2 | IDC | II | IIB | +++ | ACT+TRA | 0.0 | R |
| 122 | 50 | pre | 2.5 | IDC | III | IIB | --- | ACT | 0.2 | R |
| 123 | 46 | pre | 2.6 | IDC | II | IIA | +++ | FECD+TRA | 0.2 | R |
| 124 | 60 | post | 6.0 | IDC | II | IIIB | --+ | ACT+TRA | 0.3 | R |
| 125 | 57 | post | 3.3 | IDC | N/A | IIB | --+ | FECD+TRA | 0.0 | R |
| 126 | 54 | post | 3.7 | IDC | N/A | IIA | +-+ | ACT | 0.0 | R |
| 127 | 67 | post | 2.5 | IDC | III | IIA | +++ | ACT+TRA | 1.4 | R |
| 128 | 55 | post | 2.7 | IDC | III | IIB | --- | ACT | 2.5 | R |
| 129 | 51 | post | 3.5 | IDC | III | IIB | +++ | ACT+TRA | 1.2 | R |
| 130 | 45 | post | 4.3 | IMC | II | IIA | ++- | FECD | 1.9 | R |
| 131 | 45 | post | 3.2 | IMC | III | IIB | --- | ACT | 3.3 | NR |
| 132 | 56 | post | 2.9 | ILC | III | IIB | ++- | FECD | 2.2 | NR |
| 133 | 42 | post | 2.1 | IDC | III | IIA | +++ | FECD+TRA | 0.0 | R |
| 134 | 38 | post | 1.9 | IDC | III | IIA | --+ | ACT+TRA | 0.8 | R |
| 135 | 36 | post | 2.9 | IDC | III | IIA | +++ | FECD+TRA | 0.1 | R |
| 136 | 27 | post | 11.3 | IDC | II | IIIC | +++ | ACT+TRA | 2.5 | R |
| 137 | 36 | post | 5.1 | IDC | III | IIIC | --- | ACT | 2.5 | R |
| 138 | 66 | post | 0.8 | IDC | I | IIIC | ++- | ACT | 0.0 | R |
| 139 | 53 | post | 3.9 | IDC | N/A | IIIC | ++- | ED | 3.7 | NR |
| 140 | 62 | post | 3.5 | IDC | I | IIB | ++- | FECD | 0.0 | R |

**Supplementary Table 1 continue…**

| 141 | 63 | post | 3.7 | IDC | III | IIIC | --- | ACT | 0.0 | R |
| --- | --- | --- | --- | --- | --- | --- | --- | --- | --- | --- |
| 142 | 81 | post | 3.8 | IDC | III | IIIC | --- | DC | 9.0 | NR |
| 143 | 55 | post | 3.4 | IMC | III | IIA | --- | ACT | 1.5 | R |
| 144 | 47 | peri | 3.9 | IDC | III | IIIC | --- | ACT | 2.5 | R |
| 145 | 31 | post | 4.1 | IDC | III | IIIA | ++- | ACT | 1.2 | R |
| 146 | 34 | post | 4.1 | IDC | III | IIB | ++- | FECD | 3.0 | R |
| 147 | 71 | post | 3.6 | IDC | III | IIA | --- | ACT | 3.0 | R |
| 148 | 46 | peri | 11.6 | ILC | III | IIIA | +-+ | FECD | 9.8 | R |
| 149 | 47 | post | 3.0 | IDC | III | IIA | +-- | ACT | 2.7 | NR |
| 150 | 34 | post | 1.9 | IDC | III | IIA | --- | DC | 1.3 | R |
| 151 | 67 | post | 1.9 | IDC | III | IIIB | --- | ACT | 0.0 | R |
| 152 | 51 | peri | 1.9 | IDC | III | IIB | --- | ACT | 2.5 | NR |
| 153 | 37 | peri | 4.4 | IDC | III | IIIC | ++- | FECD | 2.5 | R |
| 154 | 47 | peri | 6.7 | IDC | III | IIB | ++- | ACT | 3.0 | R |
| 155 | 50 | peri | 1.9 | IDC | III | IIB | +-- | ACT | 1.3 | R |
| 156 | 50 | peri | 6.1 | IDC | II | IIIC | ++- | FECD | 5.3 | R |
| 157 | 66 | peri | 1.9 | IDC | N/A | IIA | --+ | FECD+TRA | 0.0 | R |
| 158 | 67 | post | 4.0 | IDC | II | IIB | --+ | FECD+TRA | 1.2 | R |
| 159 | 60 | post | 2.4 | IDC | II | IIB | +-- | FECD | 7.2 | R |
| 160 | 43 | peri | 5.3 | IDC | III | IIIC | --+ | ACT+TRA | 0.0 | R |
| 161 | 45 | peri | 2.7 | IDC | III | IIB | --+ | ACT+TRA | 2.5 | R |
| 162 | 49 | peri | 2.5 | IDC | III | IIB | +++ | ACT+TRA | 2.5 | NR |
| 163 | 50 | peri | 10.7 | IDC | II | IIIA | +++ | ACT+TRA | 2.4 | R |
| 164 | 51 | peri | 4.0 | IDC | III | IIB | ++- | FECD | 2.0 | R |
| 165 | 40 | peri | 5.9 | IDC | II | IIIA | ++- | FECD | 6.0 | R |
| 166 | 72 | post | 3.5 | IDC | III | IIB | +++ | FECD+TRA | 0.0 | R |
| 167 | 51 | peri | 3.3 | IDC | III | IIB | ++- | ACT | 5.5 | R |
| 168 | 63 | post | 4.0 | IDC | III | IIIC | --- | ACT | 0.5 | R |
| 169 | 56 | post | 3.5 | IDC | III | IIA | +-- | DC | 1.9 | R |

**Supplementary Table 1 continue…**

| 170 | 49 | pre | 4.9 | IDC | II | IIA | +++ | FECD+TRA | 2.5 | NR |
| --- | --- | --- | --- | --- | --- | --- | --- | --- | --- | --- |
| 171 | 50 | pre | 3.0 | IDC | III | IIIA | ++- | ACT | 3.2 | NR |
| 172 | 60 | post | 7.3 | IDC | II | IIIA | --- | ACT | 4.0 | R |
| 173 | 58 | post | 6.3 | IDC | III | IIIA | --+ | FECD+TRA | 0.9 | R |
| 174 | 53 | pre | 6.4 | IDC | N/A | IIIA | --+ | FECD+TRA | 0.3 | R |
| 175 | 41 | pre | 7.5 | IDC | III | IIIA | +-- | ACT | 2.0 | R |
| 176 | 72 | post | 4.7 | IDC | III | IIA | --- | ACT | 3.5 | NR |
| 177 | 53 | post | 3.1 | IDC | III | IIA | --- | ACT | 1.5 | R |
| 178 | 63 | post | 7.4 | IDC | II | IIIB | ++- | FECD | 7.5 | NR |
| 179 | 64 | post | 3.4 | IDC | II | IIB | +++ | FECD+TRA | 1.7 | R |
| 180 | 71 | post | 1.7 | IDC | III | IIA | +-+ | ACT+TRA | 0.0 | R |
| 181 | 43 | post | 5.6 | IDC | III | IIIA | --- | ACT | 1.8 | R |
| 182 | 80 | post | 2.5 | IDC | III | IIIA | --- | ACT | 4.0 | R |
| 183 | 37 | pre | 12.0 | IMC | II | IIIA | ++- | FECD | 8.0 | R |
| 184 | 27 | pre | 1.4 | IDC | III | IIIC | --- | ACT | 1.5 | R |
| 185 | 32 | pre | 2.6 | IDC | II | IIB | ++- | FECD | 4.6 | NR |
| 186 | 55 | Post | 2.0 | IDC | II | IIB | +-- | ACT | 1.0 | R |
| 187 | 60 | post | 3.2 | IDC | III | IIIC | --- | ACT | 2.5 | NR |
| 188 | 58 | Post | 3.2 | IDC | III | IIB | ++ | ACT | 3.5 | NR |
| 189 | 51 | Pre | 4.1 | IDC | III | IIIA | ++ | ACT | 1.6 | R |
| 190 | 56 | Post | 5.2 | IMC | III | IIIC | +- | ACT | 0.0 | R |
| 191 | 59 | Post | 3.1 | IDC | III | IIIC | --+ | ACT | 0.0 | R |
| 192 | 35 | Pre | 10.0 | IDC | III | IIB | ++- | ACT | 0.0 | R |
| 193 | 74 | Post | 4.5 | IDC | III | IIIA | --- | ACT | 1.4 | R |
| 194 | 58 | Post | 3.0 | IDC | III | IIB | --- | ACT | 3.4 | NR |
| 195 | 41 | Pre | 1.8 | IDC | III | IIIC | +-- | ACT | 0.0 | R |
| 196 | 57 | Post | 7.6 | IDC | III | IIIC | --- | ACT | 5.0 | NR |
| 197 | 46 | Peri | 6.0 | IDC | III | IIIA | +-- | ACT | 3.5 | R |
| 198 | 56 | Post | 4.1 | ILC | II | IIB | ++- | ACT | 3.0 | NR |

**Supplementary Table 1 continue…**

| 199 | 45 | Pre | 3.4 | IDC | II | IIB | -+- | ACT | 3.5 | NR |
| --- | --- | --- | --- | --- | --- | --- | --- | --- | --- | --- |
| 200 | 37 | Pre | 3.9 | IDC | II | IIB | ++- | ACT | 1.3 | R |
| 201 | 52 | Post | 3.4 | IDC | II | IIIA | ++- | ACT | 1.5 | R |
| 202 | 66 | Post | 2.9 | IDC | II | IIIA | ++- | ACT | 1.0 | R |
| 203 | 57 | Post | 3.1 | ILC | III | IIB | ++- | ACT | 1.9 | R |
| 204 | 44 | Pre | 1.8 | IDC | II | IIIC | ++- | ACT | 1.8 | NR |
| 205 | 35 | Pre | 2.4 | IDC | II | IIB | +-- | ACT | 1.0 | R |
| 206 | 49 | Pre | 6.1 | IDC | III | IIIA | +-- | ACT | 0.0 | R |
| 207 | 28 | Pre | 2.7 | IDC | III | IIB | --- | CIS | 2.0 | NR |
| 208 | 33 | pre | 4.5 | IDC | N/A | IIIA | ++- | FECD | 1.5 | R |

ILC: invasive lobular carcinoma, IDC: invasive ductal carcinoma, IMC: invasive micropapillary carcinoma, N/A: not available, ACT: Adriamycin and Cytoxan + Taxotere, FECD: Fluorouracil, epirubicin and cyclophosphamide + docetaxel, TC: Taxotere and cyclophosphamide, TRA: trastuzumab, D: docetaxel, XRT: radiation treatment, DC: docetaxel and cyclophosphamide, DCB: docetaxel and carboplatin, ED: epirubicin and docetaxel, CIS: cisplatin, R: Responder, and NR: Non-responder.

**Supplementary Table 2a: Classification performance for KNN model based on various type of feature sets using leave-one-out cross validation.**

| **Feature set** | **Sens**  **[%]** | **Spec**  **[%]** | **Acc**  **[%]** | **PPV**  **[%]** | **NPV**  **[%]** | **AUC** |
| --- | --- | --- | --- | --- | --- | --- |
| Feature Set I | 66 | 71 | 70 | 40 | 88 | 0.67 |
| Feature Set II | 66 | 67 | 67 | 37 | 87 | 0.65 |
| Feature Set III | 23 | 75 | 63 | 22 | 77 | 0.49 |
| Feature Set IV | 68 | 70 | 69 | 40 | 88 | 0.69 |
| Feature Set V | 64 | 74 | 72 | 42 | 88 | 0.69 |
| **Feature Set VI** | **74** | **80** | **79** | **52** | **91** | **0.76** |
| Feature Set VII | 74 | 74 | 74 | 45 | 91 | 0.74 |

**Sens:** Sensitivity; **Spec:** Specificity; **Acc:** Accuracy; **PPV:** Positive Predictive Value; **NPV:** Negative Predictive Value; **AUC:** Area under Curve.

**Feature Set I**: QUS + Texture + Core-to-Margin;

**Feature Set II**: Texture Derivative;

**Feature Set III**: Molecular Subtype;

**Feature Set IV**: QUS + Texture + Core-to-Margin;

**Feature Set V**: QUS + Texture + Molecular Subtype;

**Feature Set VI**: Texture Derivative + Molecular Subtype;

**Feature Set VII**: QUS + Texture + Core-to-Margin + Texture Derivative + Molecular Subtype;

**Table 2b: Classification performance for SVM-RBF model based on various type of feature sets using leave-one-out cross validation.**

| **Feature set** | **Sens**  **[%]** | **Spec**  **[%]** | **Acc**  **[%]** | **PPV**  **[%]** | **NPV**  **[%]** | **AUC** |
| --- | --- | --- | --- | --- | --- | --- |
| Feature Set I | 81 | 75 | 76 | 48 | 93 | 0.84 |
| Feature Set II | 81 | 76 | 77 | 50 | 93 | 0.82 |
| Feature Set III | 89 | 64 | 70 | 42 | 95 | 0.87 |
| Feature Set IV | 80 | 80 | 80 | 54 | 93 | 0.83 |
| Feature Set V | 79 | 77 | 77 | 50 | 93 | 0.81 |
| **Feature Set VI** | **79** | **86** | **85** | **63** | **93** | **0.87** |
| Feature Set VII | 87 | 81 | 83 | 58 | 96 | 0.87 |

**Sens:** Sensitivity; **Spec:** Specificity; **Acc:** Accuracy; **PPV:** Positive Predictive Value; **NPV:** Negative Predictive Value; **AUC:** Area under Curve.

**Feature Set I**: QUS + Texture + Core-to-Margin;

**Feature Set II**: Texture Derivative;

**Feature Set III**: Molecular Subtype;

**Feature Set IV**: QUS + Texture + Core-to-Margin;

**Feature Set V**: QUS + Texture + Molecular Subtype;

**Feature Set VI**: Texture Derivative + Molecular Subtype;

**Feature Set VII**: QUS + Texture + Core-to-Margin + Texture Derivative + Molecular Subtype;

**Table 3a: Average classification performance for KNN and SVM-RBF models from hold-out cross validation with 20% for testing and 80% for training of data set.**

| **Classifier** | **Data set** | **Sensitivity [%]** | **Specificity [%]** | **Accuracy [%]** | **AUC** |
| --- | --- | --- | --- | --- | --- |
| KNN | Training set | 68 ± 1 | 71 ± 9 | 70 ± 7 | 0.71 ± 0.07 |
|  | Test set | 68 ± 7 | 67 ± 6 | 68 ± 4 | 0.69 ± 0.02 |
| SVM | Training set | 81 ± 4 | 65 ± 2 | 68 ± 2 | 0.73 ± 0.04 |
|  | Test set | 69 ± 5 | 78 ± 6 | 71 ± 4 | 0.71 ± 0.04 |

**Table 3b: Average classification performance for KNN and SVM-RBF models from hold-out cross validation with 10% for testing and 90% for training of data set.**

| **Classifier** | **Data set** | **Sensitivity [%]** | **Specificity [%]** | **Accuracy [%]** | **AUC** |
| --- | --- | --- | --- | --- | --- |
| KNN | Training set | 74 ± 1 | 78 ± 9 | 77 ± 3 | 0.76 ± 0.05 |
|  | Test set | 82 ± 7 | 75 ± 5 | 81 ± 2 | 0.08 ± 0.03 |
| SVM | Training set | 68 ± 9 | 70 ± 3 | 70 ± 1 | 0.72 ± 0.03 |
|  | Test set | 70 ± 3 | 75 ± 3 | 71 ± 3 | 0.71 ± 0.07 |
